# Nutrition delivery after emergency laparotomy in surgical ward: a retrospective cohort study

**DOI:** 10.1007/s00068-021-01659-3

**Published:** 2021-04-02

**Authors:** Juho Nurkkala, Sanna Lahtinen, Aura Ylimartimo, Timo Kaakinen, Merja Vakkala, Marjo Koskela, Janne Liisanantti

**Affiliations:** Medical Research Centre and Research Group of Surgery, Anesthesia and Intensive Care, Department of Anesthesiology, University of Oulu, Oulu University Hospital, P.O. Box 21, 90029 Oulu, Finland

**Keywords:** Nutrition adequacy, Nutritional support, Parenteral nutrition, Enteral nutrition, Emergency laparotomy

## Abstract

**Purpose:**

Adequate nutrition after major abdominal surgery is associated with less postoperative complications and shorter hospital length of stay (LOS) after elective procedures, but there is a lack of studies focusing on the adequacy of nutrition after emergency laparotomies (EL). The aim of the present study was to investigate nutrition adequacy after EL in surgical ward.

**Methods:**

The data from 405 adult patients who had undergone emergency laparotomy in Oulu University Hospital (OUH) between years 2015 and 2017 were analyzed retrospectively. Nutrition delivery and complications during first 10 days after the operation were evaluated.

**Results:**

There was a total of 218 (53.8%) patients who were able to reach cumulative 80% nutrition adequacy during the first 10 postoperative days. Patients with adequate nutrition (> 80% of calculated calories) met the nutritional goals by the second postoperative day, whereas patients with low nutrition delivery (< 80% of calculated calories) increased their caloric intake during the first 5 postoperative days without reaching the 80% level. In multivariate analysis, postoperative ileus [4.31 (2.15–8.62), *P* < 0.001], loss of appetite [3.59 (2.18–5.93), *P* < 0.001] and higher individual energy demand [1.004 (1.003–1.006), *P* = 0.001] were associated with not reaching the 80% nutrition adequacy.

**Conclusions:**

Inadequate nutrition delivery is common during the immediate postoperative period after EL. Oral nutrition is the most efficient way to commence nutrition in this patient group in surgical ward. Nutritional support should be closely monitored for those patients unable to eat.

**Trial registration number:**

Not applicable.

## Introduction

Postoperative nutrition is considered as standard care after major abdominal surgery [1]. Adequate nutrition after abdominal surgery has been reported to reduce postoperative complications and shorten hospital length of stay (LOS). Wound and tissue healing processes following the surgical insult depend highly on adequate nutrition [1]. During the past decade “enhanced recovery after surgery” (ERAS) programs have been introduced for patients undergoing elective surgical operations [1, 2]. Although originally ERAS was designed for elective surgical settings, recently it has also been utilized for patients recovering from emergency surgery [3–5]. One key component of ERAS protocols is perioperative nutrition delivery [3]. In addition of postoperative nutritional care, ERAS pathway includes preoperative nutrition optimization for patients with low nutritional status since those patients are known to been predispose to postoperative complications [6]. In emergency setting, significance of postoperative nutrition cannott be overstated because preoperative adjusting of nutritional status might be impossible, and preceding malnutrition impairs later metabolic recovery [6]. Therefore, early evaluation of baseline nutritional status is important.

Early oral nutrition is recommended generally for all surgical patients but in cases oral nutrition is not tolerated, nutrition should be conducted by nutritional support [1, 7]. Most studies evaluating adequate nutrition after abdominal surgery have been conducted in elective patient settings, but the adequacy of postoperative nutrition and its impact on short-term outcome after emergency laparotomy (EL) are rarely described [7, 8]. Although the concept of postoperative nutritional care has been introduced lately also among EL patients [9], there is a paucity of studies evaluating the adequacy of postoperative in-hospital nutrition in this patient group [8]. Previous studies focus mainly on the optimal initiation of oral intake and not on the actual caloric intake during the recovery phase [10, 11]. The aim of the present study was to evaluate the adequacy of nutrition in surgical ward after EL during the immediate postoperative period and to investigate the factors associated with adequate nutrition delivery.

## Methods

The present study is an observational retrospective single center study performed in Oulu University Hospital, Finland. The study design was approved by the hospital administration (journal number 66/2018). Due to the retrospective study setting and according to the local regulations, no statement from the local ethics committee was obtained.

### Patients

We included all adult (> 18 years) patients undergone EL between the years 2015 and 2017 in Oulu University Hospital to the study. Patients with an admission to the intensive care unit for longer than first postoperative day were excluded. There was a total of 460 patients meeting the inclusion criteria but 55 of those were excluded due to missing data, leaving 405 patients into the final analysis.

### Nutrition

We assessed the patient’s nutrition between the days 1 and 10 following the surgery. Calories administered via nutritional support were obtained from the medical records by calculating the received daily amounts of intravenous dextrose as well as parenteral and enteral nutrition. Calories received via oral route were determined from the patient records by calculating the daily food consumption in milliliters and approximating the daily content of calories based on the average hospital diet (1800 kcal/day in OUH). In this study, “oral intake” refers to normal peroral eating whereas “enteral nutrition” refers to enteral tube feeding conducted via nasogastric tube. Daily caloric demand was estimated as 30 kcal/ideal body weight (IBW) which was derived from the ESPEN guidelines for surgical patients [1]. Ideal body weight was calculated as the Devine formula for men and the Robinson formula for women [12]. Patient’s individual cumulative caloric count was obtained by adding all administered oral intake, EN, PN and dextrose–calories from the follow-up period. Nutrition adequacy percentage was calculated for each patient with following pattern: (Follow up period’s cumulative caloric count [Kcal]/(Length of stay[d] * IBW [Kg] * 30 [Kcal/(kg*d)] * 100). Previous studies among patients undergoing elective major abdominal surgery report that nutrition adequacy reaches 80% of estimated individual need during the immediate postoperative period [2, 13]. Accordingly, we determined the nutrition adequacy of 80% as primary endpoint for the present study. Preoperative malnutrition was evaluated using the nutrition related index (NRI) presented by Parhar et al. [12] and patients with NRI less than 97.5 were considered as preoperatively malnourished. Nausea, gastric pain or loss of appetite was recorded when the patient at least once refused to consume meal due to any of these reasons. To clarify the interpretation of results, we defined the patients who received more than 80% of calculated energy demand as “group adequate” and patients who received less than 80% of calculated energy demand as “group low”. Metabolic recovery was evaluated from the laboratory results recorded on the second postoperative day by forming CRP/albumin ratio.

Postoperative complications during the hospital stay were detected from the medical records. Complications were categorized as surgical and medical. Fascial dehiscence, wound infection, wound bleeding, seroma, anastomotic leak, intra-abdominal abscess and ileus were considered as operative complications whereas respiratory dysfunction, pneumonia, pulmonary embolus, transient ischemic attack (TIA), high-output stoma, kidney dysfunction, liver dysfunction, cardiopulmonary resuscitation, atrial fibrillation (FA), and sepsis were considered as medical complications. Respiratory dysfunction was recorded in cases ventilation or oxygenation deficit occurred.

### Statistical analysis

IBM SPSS Statistics 25 software (IBM SPSS Statistics for Windows, Version 25.0, Armonk, NY, USA) was used to perform statistical analyses. Categorical variables are expressed as numbers (*n*) and percentages (%) whereas continuous variables are expressed as medians and 25–75th percentiles [25–75th PCT]. Categorical variables were tested using the Pearson’s Chi-square and the continuous variables were tested using the Mann–Whitney test. Two-tailed *P *values below 0.05 were considered statistically significant. Logistic regression analysis was performed to calculate OR for not reaching the 80% nutrition adequacy cut-off value. Age and gender as well as continuous and categorial variables with univariate significance < 0.1 were included one by one using the enter method. The factors with *P* value < 0.05 were kept in the model, as well as those with significant impact on the log-likelihood function.

## Results

There was a total of 218 (53.8%) patients who reached 80% nutrition adequacy during the postoperative follow-up period (group adequate). The most common admission diagnosis in both groups was bowel obstruction (98 (45.0) vs 76 (40.6), *P* = 0.382). The admission diagnosis had no impact on reaching the limit of 80% nutrition adequacy. Patients in the group adequate were younger, more often female, had lower weight and lower ideal body weight (IBW) and had malignancies less often than the patients in the group low. The group adequate had a shorter hospital length of stay (LOS) (8 [5–12] vs 10 [6–14], *P* = 0.002) and shorter postoperative LOS (6 [4–8] vs 7 [5–11], *P* < 0.001) than the group low. The group adequate were also more likely to get discharged alive although the number of in-hospital deaths was small. The rate of preoperatively malnourished patients was comparable between the groups. There were no differences in other variables on patient demographics between the study groups (Table 1).

**Table 1:** Patient demographics

	Group adequate *N* = 218	Group low *N* = 187	*P* value
Age	66.5 (51.8–76.0)	67.0 (54.0–78.0)	< 0.001
Male gender	85 (39.0)	120 (64.2)	< 0.001
Weight (kg)	70 (60–82)	73 (65–85)	0.032
IBW	58 (54–66)	67 (57–73)	< 0.001
ASA	3 (2, 3)	3 (2–4)	0.063
CCI	4 (2–6)	4 (2–7)	0.233
Admission diagnosis			
Bowel obstruction	98 (45.0)	76 (40.6)	0.382
Peritonitis	29 (13.3)	26 (13.9)	0.860
Bowel ischemia	8 (3.7)	14 (7.5)	0.091
Ventricular or duodenal ulcer	7 (3.2)	5 (2.7)	0.715
Tumor	24 (11.0)	27 (14.4)	0.300
Re-operation	28 (12.8)	23 (12.3)	0.869
Other	24 (11.0)	16 (8.6)	0.409
Malignancy	73 (33.5)	87 (46.5)	0.007
Previous GI surgery			
None	87 (39.9)	84 (44.9)	0.331
During current admission	23 (10.6)	24 (12.8)	
Before current admission	108 (49.5)	79 (42.2)	
Preoperative CRP	34 (7–135)	48 (9–153)	0.225
Preoperative leukocyte count	10 (7–13)	10 (7–13)	0.901
Administered antibiotics	148 (67.9)	131 (70.1)	0.639
NRI less than 97,5	79 (36.2)	67 (35.8)	0.718
Postoperative LOS (d)	6 (4–8)	7 (5–11)	< 0.001
Hospital LOS (d)	8 (5–12)	10 (6–14)	0.002
In-hospital death	1 (0.5)	11 (5.9)	< 0.001

Values are numbers (percentage) or medians (25–75th percentiles)

*IBW* Ideal body weight, *ASA* American Society of Anesthesiologists classification, *CCI* Charlson comorbidity index, *GI* gastro-intestinal, *CRP* C-reactive protein, *NRI* nutrition-related index, *LOS* length of stay

**Fig. 1:**
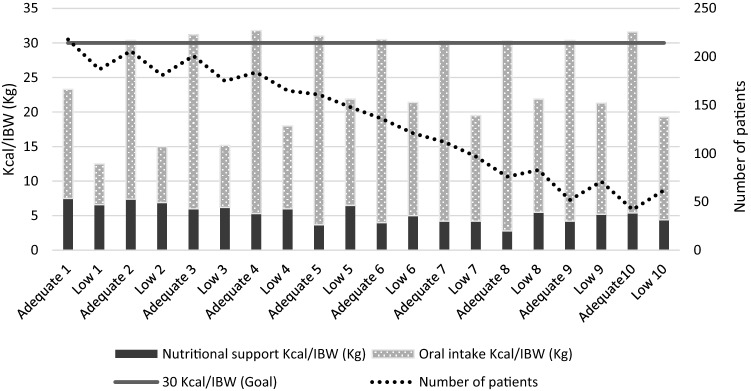
The median daily delivery of nutritional support and oral intake. The values are presented for the group adequate and for the group low separately for each follow-up day. Nutritional support and oral intake bars are median daily administered kilocalories divided by patient’s individual ideal body weight (IBW in kilograms). Nutrition goal is 30 kcal/IBW (kg). The number of patients refers to the daily number of patients in the ward on each follow-up day

The patients in the group adequate reached the required 80% level of nutrition demand starting from the second postoperative day. However, the patients in the group low never reached that level although the amount of administered energy slightly increased during the first half of the follow-up period. The amount of administered nutritional support did not increase during the follow-up period in the group low, although the oral intake remained low (Fig. 1).

The patients in the group adequate had smaller calculated daily energy demand [1745 kcal (1608–1978) vs 2005 (1706–2195), *P* < 0.001], received less parenteral nutrition [42 kcal (0–233) vs 125 (0–277), *P* = 0.014] and were administered more oral calories [1440 kcal (1238–1710) vs 836 (540–1080), *P* < 0.001] than in the group low. There was no difference in the number of patients receiving enteral nutrition [2 (0.9) vs 3 (1.6), *P* = 0.533] between the study groups. The patients in the group low suffered more often from loss of appetite compared to the patients in the group adequate [102 (54.5) vs 66 (30.3), *P* < 0.001]. They also had higher CRP/albumin ratio in the second postoperative day [8.4 (5.0–12.4) vs 6.4 (4.1–10.6), *P* = 0.024]. There were no differences nausea or gastric pain between the study groups, although the incidence was high in both groups (Table 2).

**Table 2:** Nutritional characteristics of patients

	Group adequate *N* = 218	Group low *N* = 187	*P* value
Calculated daily energy demand	1745 (1608–1978)	2005 (1706–2195)	< 0.001
Cumulative daily calories	1753 (1530–1890)	1138 (711–1360)	< 0.001
Administered daily 5% dextrose (Kcal)	200 (120–326)	189 (100–282)	0.200
Administered daily Pn (Kcal)	42 (0–233)	125 (0–277)	0.014
Administered daily oral intake (kcal)	1440 (1238–1710)	836 (540–1080)	< 0.001
Nausea or gastric pain	85 (39.0)	62 (33.2)	0.438
Loss of appetite	66 (30.3)	102 (54.5)	< 0.001
Dietician evaluation	17 (7.8)	9 (4.8)	0.282

Values are numbers (percentage) or medians (25–75th percentiles)

*Pn* parenteral nutrition, *En* enteral nutrition

Surgical complications were recorded more often in the group low [91 (48.7) vs 78 (35.8), *P* = 0.009] whereas there was no difference in the incidence of medical complications between the study groups. The patients in the group low had more often pneumonia, ileus, and kidney dysfunction. High-output stoma occurred more often in the group adequate, although the incidence was low (Table 3).

**Table 3:** Postoperative complications

	Group adequate *N* = 218	Group low *N* = 187	*P* value
Surgical complications	78 (35.8)	91 (48.7)	0.009
Fascial dehiscence	6 (2.8)	12 (6.4)	0.074
Wound infection	18 (8.3)	20 (10.7)	0.401
Wound bleeding	5 (2.3)	6 (3.2)	0.572
Seroma	7 (3.2)	4 (2.1)	0.508
Anastomotic leak	1 (0.5)	5 (2.7)	0.066
Intra-abdominal abscess	11 (5.0)	15 (8.0)	0.228
Ileus	19 (8.7)	53 (28.3)	< 0.001
Re-operation	18 (8.3)	20 (10.7)	0.401
Medical complications	58 (26.6)	63 (33.7)	0.120
Respiratory dysfunction	18 (8.3)	24 (12.8)	0.127
Pneumonia	15 (6.9)	27 (14.4)	0.012
Pulmonary embolus	3 (1.4)	3 (1.6)	0.850
TIA	1 (0.5)	0 (0.0)	0.354
High-output stoma	8 (3.7)	0 (0.0)	0.006
Kidney dysfunction	2 (0.9)	11 (5.9)	0.005
Liver dysfunction	0 (0.0)	1 (0.5)	0.218
Cardiopulmonary resuscitation	0 (0.0)	2 (1.1)	0.126
FA	2 (0.9)	2 (1.1)	0.934
Sepsis	9 (4.1)	12 (6.4)	0.300

Values are numbers (percentage) or medians (25–75th percentiles)

*TIA* transient ischemic attack, *FA* atrial fibrillation

In the logistic regression analysis, risk factors for not receiving 80% of calculated energy need were post-operative ileus [OR 4.31 (2.15–8.62), *P* < 0.001], loss of appetite [OR 3.59 (2.18–5.93), *P* < 0.001], higher daily energy demand [OR 1.004 (1.003–1.006), *P* < 0.001] and refraining of oral intake on the first postoperative day [OR 4.80 (2.73–8.44), *P* < 0.001] (Table 4).

**Table 4:** OR and 95% confidence intervals for not receiving 80% nutritional adequacy

	OR (95% Cl)	*P* value
Male gender	0.51 (0.22–1.17)	0.112
Postoperative ileus	4.31 (2.15–8.62)	< 0.001
Loss of appetite	3.59 (2.18–5.93)	< 0.001
Daily energy demand	1.004 (1.003–1.006)	< 0.001
No oral intake on the first postoperative day	4.80 (2.73–8.44)	< 0.001

## Discussion

The main finding of the present study was that only 53.8% of the patients received 80% of their calculated energy demand. Early oral intake was associated with better nutrition adequacy, whereas in most cases nutritional support did not provide enough calories for patients unable to eat. To our knowledge, this is the first study evaluating the adequacy of postoperative nutrition in surgical ward after EL.

It has been reported previously that early oral nutrition after major abdominal surgery is safe in both elective and emergency settings [1, 7, 10]. Moreover, according to previous reports, initiating oral intake in the first postoperative day after elective colorectal surgery reduces complications and shortens hospital LOS in both ERAS and conventional settings of recovery [4, 14, 15]. Our results also suggest that oral intake should be initiated in the very beginning of the recovering process because early oral intake was associated with better nutrition adequacy than nutritional support. Furthermore, we think that early oral intake after EL might be even more essential than after elective surgery, because preoperative optimizing of patient’s nutritional status before EL is challenging or even impossible. In the present study, the patients with higher nutrition adequacy had a shorter hospital LOS. This was an expected finding since adequate nutrition is associated with shortened LOS also in an elective setting [7]. Patients in the group low had higher CRP/albumin ratio in the second postoperative day. We think that this might indicate that patients with low nutrition adequacy are predisposed to prolonged metabolic recovery, or more severe inflammation predisposes to low level of postoperative nutrition, but any conclusions about causal relationship of this phenomenon should be make with caution.

In the present study, nutritional support provided only a minor share of administered nutrition. Furthermore, administered calories via nutritional support remained low throughout the entire follow-up period. In the intensive care setting, it has been previously reported that nutrition adequacy can be as low as 26–32% of calculated demand when nutrition is provided solely by nutritional support [16]. In our study, nutritional support’s contribution to overall nutrition provision was even lower. A reason for this might be that nutritional support is more common in the ICU setting than in surgical ward since the majority of ICU patient’s nutrition is commenced by nutritional support [17]. Furthermore, hyperosmolar intravenous nutrition solutions require central venous catheters which are not routinely used in general surgical ward. In the present study, the amount of calories provided by nutritional support did not rise throughout the follow-up. We hypothesize that in many cases the attending physician may have estimated that oral intake would be achieved soon and nutritional support would not be needed. For achieving adequate level of nutrition, nutritional support volumes should be gradually increased for patients unable to digest food during the first days of recovery. In the present study, incidence of preoperative malnutrition was more than 33% within both groups. According to our results, special attention should be made to preceding nutritional status on the ward after the EL, because role of nutritional support is even more evident among patients with poor baseline nutritional status and ERAS pathways cannott be achieved preoperatively in the emergency setting [1]. In studies contemplating ERAS protocols in elective patient settings, the amount of provided calories via nutritional support has been enhanced using multidisciplinary teams and specified nutrition protocols [18]. These methods might enhance nutritional support also in EL patients. In this cohort, only few patients received enteral nutrition. Based on our findings, one could hypothesize that postoperative “Nutrition treatment bundle” could be routinely implemented for patients with preceding malnutrition as well as for patients with no oral intake in the first postoperative day. This could include routine consultation of nutrition therapist, routine insertion of nasogastric tube, bolus enteral nutrition and advantage of enriched energy rich enteral solutions. Bolus enteral nutrition could be included to the proposed protocol since the amount of administered enteral nutrition has been increased by providing intermittent boluses rather than continuous infusions in the ICU setting [19].

According to our results, postoperative ileus and loss of appetite were major risk factors for not reaching 80% nutrition adequacy. The prevalence of postoperative ileus was in line with previous findings in the literature [20]. ERAS protocols promote early initiation of oral nutrition to prevent postoperative ileus [20]. According to our results, the concept of early oral nutrition may be generalized also to patients recovering from EL since patients in the group adequate had a lower incidence of ileus and correspondingly they started oral nutrition earlier. It has been previously reported that the duration of postoperative ileus could be shortened after elective colorectal surgery by providing patients their favorite food and chewing gum and even by presenting them with food-related programs [13, 20]. Accordingly, among general hospital population, nutrition adequacy has increased by paying attention to food taste and by introducing postponed and more individual meal times for patients [18, 21]. These might also be useful implementations after EL. In the present study, malignancy was associated with low nutrition adequacy. It has been demonstrated previously that tumors can secrete inflammatory markers which may impair appetite [12]. According to our results, EL patients with malignancy are particularly predisposed to postoperative underfeeding.

We report that nutritional support should be focused for patients whose oral intake is inadequate or nonexistent. In these situations, it might be possible to achieve better nutrition adequacy by increasing parenteral nutrition, but the effect of added parenteral nutrition on postoperative outcome remains unknown. The role of parenteral nutrition in immediate postoperative period is still partly controversial [1, 17], but this study suggests close monitoring of nutritional support in surgical ward to increase nutrition adequacy.

### Limitations

This study has several limitations. This is a retrospective cohort study with all the known risks of bias, which are obvious as our study population is reasonably heterogenous as we included all EL patients to the study. We used logistic regression analysis to mitigate this. Moreover, our patient population mimics accurately the situation of surgical ward with unselected patients. It would have also been interesting to include ICU patients in the study but since ICU’s nutrition guidelines differ considerably from the guidelines of surgical ward, the comparison would have been quite difficult [1, 22]. In this retrospective study, we had to calculate consumed oral intake calories from the patient records, which may lead to inaccurate caloric count. Precise data of consumed oral calories would require a new study with a prospective setting.

### Clinical significance

By conducting this longitudinal cohort study, we wanted to assess the level of nutrition adequacy after EL in surgical ward since actual success of postoperative nutrition in this patient group has not been reported previously. Our results show that inadequate nutrition after EL is a common problem. Oral intake should be initiated in the beginning of recovering period since it seems to be the most effective way to obtain a sufficient level of postoperative nutrition. Special emphasis should be put to avoiding and mitigating ileus and efforts should be made to improve appetite. For patients whose nutrition is commenced mainly or solely by nutritional support, regular central venous catheter insertion, closer monitoring of nutrition delivery and utilization of multidisciplinary nutrition teams might improve the adequacy of nutritional support in this patient group [23].

## Conclusion

Adequate nutrition after EL should be pursued actively since it was associated with a smaller amount of complications and shorter hospital LOS than inadequate caloric intake. Eating is the most efficient method to provide nutrition after EL in surgical ward. According to our results, efforts should be made to initiate postoperative oral nutrition as soon as possible as previous literature on elective surgery also suggests [1]. Nutritional support should be used under close monitoring because it appears to be insufficient in this patient group.
